# Association between bone mineral content and sarcopenia in hospitalized patients with type 2 diabetes mellitus: a cross-sectional study

**DOI:** 10.3389/fmed.2026.1956269

**Published:** 2026-09-15

**Authors:** Wei Yan, Jun-Xia Han, Wen-Lu Zhao, Yong-Qiang Yang

**Affiliations:** 1 Department of Geriatric Medicine, the First Affiliated Hospital of Soochow University, Suzhou, Jiangsu, China; 2 Department of Endocrinology, the First Affiliated Hospital of Soochow University, Suzhou, Jiangsu, China; 3 Department of Radiology, The Second Hospital of Soochow University, Suzhou, Jiangsu, China; 4 State Key Laboratory of Radiation Medicine and Protection, Soochow University, Suzhou, Jiangsu, China; 5 Department of Abdominal and Pelvic Tumor Treatment, The Second Affiliated Hospital of Soochow University, Suzhou, Jiangsu, China; 6 Department of Radiotherapy & Oncology, The Second Affiliated Hospital of Soochow University, Suzhou, Jiangsu, China

**Keywords:** biomarker, bone mineral content, geriatric medicine, sarcopenia, type 2 diabetes mellitus

## Abstract

**Purpose:**

Type 2 diabetes mellitus (T2DM) increases the risk of sarcopenia, which independently contributes to bone fragility, especially among the elderly. Bone mineral content (BMC) is a parameter that helps in the assessment and monitoring of bone mass. This study aims to identify the correlation between BMC and sarcopenia in hospitalized patients with T2DM.

**Patients and methods:**

A total of 1,135 inpatients admitted with T2DM in this study and classified into two groups (sarcopenia and non-sarcopenia group). A comparative analysis of clinical data and body composition was performed between the two groups. A multivariable logistic regression model was used to assess the independent association of BMC with sarcopenia.

**Results:**

The sarcopenia group showed a lower BMC level than the non-sarcopenia group (*P* < 0.001). The BMC level was correlated with a history of sarcopenia (*P* < 0.001). The receiver operator characteristic (ROC) curve analysis showed a medium ability of BMC in predicting sarcopenia (area under the ROC curve=0.760). The optimal cut-off level for BMC to predict sarcopenia was found to be < 2.86 kg. Finally, in the multivariable logistic regression model, a BMC level < 2.86 kg was significantly associated with sarcopenia [odds ratio (OR) = 4.994, 95% confidence interval [CI] 3.130–7.967, *P* < 0.001]. Additional analyses revealed that lower BMC level was associated with an increased risk of sarcopenia irrespective of the subgroup of age (including elderly group and non-elderly group).

**Conclusion:**

Our results confirmed that in hospitalized patients with T2DM, BMC levels are inversely associated with sarcopenia and may therefore serve as a biomarker for sarcopenia discrimination. But additional studies are needed to confirm and further elucidate our results.

## Introduction

Type 2 diabetes mellitus (T2DM), caused by impaired insulin secretion or functional defects, is an increasingly prevalent metabolic disease worldwide (1). Sarcopenia is a progressive and generalized skeletal muscle disorder that is associated with an increased likelihood of adverse outcomes, including falls, fractures, physical disability, and mortality (2) and is preventable and treatable in the early stage (3). The prevalence of sarcopenia in patients with T2DM is increased, ranging from 7% to 29.3% (4). Older adults with T2DM are at a 1.37-fold higher risk of developing sarcopenia than those without diabetes (5). Sarcopenia contributes to the cause and consequences of T2DM (6). In particular, diabetic patients are at higher risk for sarcopenia because of insulin resistance, chronic inflammation, oxidative stress and decreased physical activity. Moreover, sarcopenia disrupts glycemic control and exacerbates the development of T2DM, resulting in various complications (7). Additionally, diabetic patients with sarcopenia are predisposed to long-term diabetes complications, frailty, hospitalizations, and premature death (8, 9). Therefore, it is urgent to explore markers to distinguish the severity of T2DM complicated with sarcopenia.

Bone mineral content (BMC) is a parameter that helps in the assessment and monitoring of bone mass, and corresponds to the amount in grams or kilograms of bone tissue (10). Notably, despite having higher bone mineral density (BMD) than non-diabetic individuals, patients with T2DM who have poor glycemic control and chronic complications exhibit an elevated fracture risk (11, 12). Because bone and muscle act as a functional unit, muscle impairment in T2DM that leads to sarcopenia also independently adds to bone fragility in these patients (13, 14). In older adults, T2DM frequently coexists with osteoporosis, sarcopenia, and fractures. Emerging data suggest that these conditions share overlapping pathophysiological mechanisms (15). Glucose, bone, and muscle metabolism are closely interconnected within a complex regulatory network (16). Although previous studies have showed that bone fragility and sarcopenia are associated in patients with T2DM, little is known about the correlation between BMC levels and sarcopenia in hospitalized patients with T2DM.

To investigate this issue, we quantified the body composition of BMC levels in patients with T2DM, who were categorized into sarcopenia and non-sarcopenia groups. And then we evaluated the association between BMC levels and sarcopenia. This study aimed to identify a biomarker capable of discriminating sarcopenia severity and to discover a possible therapeutic target for T2DM patients with sarcopenia.

## Materials and methods

### Patients

A total of 1,135 T2DM patients hospitalized in the Department of Endocrinology and Department of Geriatric Medicine of the First Affiliated Hospital of Soochow University from June 2023 to June 2024 were included in this study for analysis. Inclusion criteria: (1) meet the diagnostic criteria for T2DM issued by World Health Organization in 1999 (17); (2) cooperate to receive the evaluation of muscle mass, muscle strength, and physical functioning. Exclusion criteria: (1) age < 18 years old; (2) T1DM, gestational diabetes, or other special types of diabetes; (3) malignant tumors; (4) autoimmune diseases; (5) severe heart, liver, or kidney diseases; (6) medications that may affect body composition, such as diuretics, adrenocortical hormones, etc.; (7) edema of both lower limbs; (8) stay in bed for a long time. The study was performed in compliance with the Helsinki Declaration and received ethical clearance from the institutional review board. All participants provided written informed consent, and their clinical data were de-identified prior to the analyses.

### Data collection

Demographic data, clinical variables, laboratory results, and medication histories were retrieved from electronic medical records. Blood samples were acquired to perform biochemical analysis after 12 h of fasting. A Hitachi 7600 automatic biochemical analyzer was used to determine the biochemical markers and lipid profiles. Glycated hemoglobin (HbA1c) was quantified via high-performance liquid chromatography (Bio-Rad Laboratories, Hercules, CA), while body mass index (BMI) was derived from the standard formula of weight (kg) divided by height squared (m^2^). Waist circumference measurement: take the midpoint of the line between the lowest edge of the rib arch and the highest point of the iliacbone, horizontally surround the waist for 1 week, and measure the circumference at the end of calm exhalation. Blood pressure was recorded twice from the right upper arm after a 15-minute rest in the seated position. Systolic and diastolic pressures were averaged from the two readings. To assess the body composition of all participants, we used a bioelectrical impedance analyzer (BIA) (Inbody 770 Co., Seoul, Korea) by following the techniques, procedures, and precaution in the manufacturer's protocol. The BMC value reported by the device represents whole-body bone mineral content derived from a proprietary algorithm based on segmental impedance measurements and demographic parameters (age, sex, height, and weight). To reduce fluid-shift errors, measurements were standardized by timing (same time of day, post-absorptive state), pre-test restrictions (no exercise for ≥ 12 h, no alcohol/caffeine for ≥ 48/8 h), bladder voiding, and a 5-min seated rest prior to the 2-min upright scan. Muscle strength was evaluated by a specially assigned person using an electronic grip strength tester (TH-01, Xiangshan Company, China), with an accuracy of 0.1 kg. Physical functioning was evaluated using the 6 m walking test by a specially assigned person.

### Diagnostic criteria

Sarcopenia was diagnosed according to the 2025 consensus of the Asian Working Group for Sarcopenia (AWGS) (18), defined as concurrent low muscle mass and low muscle strength, with physical performance considered an outcome indicator. Low muscle mass (BIA-derived) was defined as SMI, that is, for age 50–64, male < 7.6 kg/m^2^, female < 5.7 kg/m^2^; for age ≥ 65, male < 7.0 kg/m^2^, female < 5.7 kg/m^2^; with additional BMI-adjusted cutoffs, male < 0.90, female < 0.63 (age 50–64) and male < 0.83, female < 0.57 (age ≥ 65). Low muscle strength (handgrip): for age 50–64, male < 34 kg, female < 20 kg; for age ≥ 65, male < 28 kg, female < 18 kg. The cutoffs for the 50–64 age group were also applied to participants aged < 50 years.

### Statistical analysis

All statistical analyses were conducted with SPSS version 26.0. Continuous variables are described as the mean ± standard deviation. Categorical variables are expressed as frequencies and percentages. The continuous variables were compared between the sarcopenia and non-sarcopenia groups using Student's t-test and the Mann–Whitney U test for variables with normal and skewed distributions respectively. Chi-square tests were applied to compare categorical variables between sarcopenic and non-sarcopenic groups. Correlations between BMC and relevant variables were assessed using Spearman's rank test. Discriminative capacity of BMC and bone metabolic markers (osteocalcin, parathyroid hormone (PTH), calcitonin, 25-hydroxyvitamin D [25(OH)D], total procollagen type I N-terminal propeptide (TP1NP), and carboxy-terminal cross-linked telopeptide of type I collagen (CTX)) for sarcopenia was assessed by receiver operating characteristic (ROC) curve analysis, and optimal cut-offs were derived from the Youden index. Multivariable logistic regression models were used to analyze the association between BMC < optimal cut-off level and sarcopenia. Odds ratios (ORs) with 95% confidence intervals (CIs) were calculated. The variables for which *P* < 0.1 was obtained in the univariate analysis were considered for use in the multivariable model.

## Results

### Patient demographics

In our study, a total of 1,135 T2DM patients were enrolled, including 528 patients (46.5%) with sarcopenia, aged 61.73 ± 14.88 years old, and 607 patients (53.5%) without sarcopenia, aged 53.75 ± 15.80 years old. The baseline characteristics of the research population with and without sarcopenia are shown in Table 1. The sarcopenia group showed a lower BMC level than the non-sarcopenia group (*P* < 0.001). Patients with sarcopenia were more likely to have osteoporosis, coronary artery disease (CAD), chronic kidney disease (CKD) and undergo oral hypoglycemic and insulin therapy. High-density lipoprotein (HDL), HbA1c, fasting insulin and 25(OH)D levels were higher, and systolic blood pressure, diastolic blood pressure, hemoglobin, albumin, prealbumin, uric acid, fasting blood glucose, alanine transaminase, aspartate aminotransferase, estimated glomerular filtration rate (eGFR), total cholesterol, triglyceride, PTH and all body composition indicators were lower in patients with sarcopenia than in the controls. Characteristics of the participants according to age and sarcopenia status are showed in Table 2. Elderly patients with sarcopenia were higher rate of CAD, and had lower systolic blood pressure, diastolic blood pressure, prealbumin, and BMC (*P* < 0.05).

**Table 1:** Baseline characteristics of the research population with and without sarcopenia.

Characteristic	Total *N* = 1135	Non-sarcopenia *N* = 607 (53.5%)	Sarcopenia *N* = 528 (46.5%)	*P* value
Age, yrs	57.47 ± 15.88	53.75 ± 15.80	61.73 ± 14.88	< 0.001
Male	737 (64.9%)	373 (61.4%)	364 (68.9%)	0.008
Smoking	229 (20.2%)	124 (20.4%)	105 (19.9%)	0.820
Drinking	151 (13.3%)	89 (14.7%)	62 (11.7%)	0.149
NAFLD	424 (37.4%)	296 (48.8%)	128 (24.2%)	< 0.001
Metabolic syndrome	801 (70.6%)	475 (78.3%)	326 (61.7%)	< 0.001
Hypertension	528 (46.5%)	281 (46.3%)	247 (46.8%)	0.870
Osteoporosis	95 (8.4%)	40 (6.6%)	55 (10.4%)	0.047
Fracture	73 (6.4%)	34 (5.6%)	39 (7.4%)	0.221
Coronary artery disease	91 (8.0%)	30 (4.9%)	61 (11.6%)	< 0.001
Chronic kidney disease	90 (7.9%)	35 (5.8%)	55 (10.4%)	0.004
Systolic blood pressure, mmHg	131.10 ± 18.44	132.32 ± 17.66	129.71 ± 19.21	0.018
Diastolic blood pressure, mmHg	83.48 ± 11.37	85.27 ± 11.26	81.42 ± 11.15	< 0.001
White blood cell, 10^9^/L	6.49 ± 1.86	6.50 ± 1.78	6.47 ± 1.95	0.776
Hemoglobin, g/L	137.02 ± 17.86	139.59 ± 17.98	134.07 ± 17.26	< 0.001
Platelets, 10^9^/L	214.41 ± 63.58	215.67 ± 61.58	212.95 ± 65.84	0.473
Hs-CRP, mg/L	3.80 ± 4.43	3.72 ± 4.03	3.88 ± 4.85	0.549
Albumin, g/L	39.41 ± 4.12	39.68 ± 3.92	39.10 ± 4.32	0.018
Prealbumin, mg/L	227.96 ± 56.28	236.89 ± 55.46	217.71 ± 55.52	< 0.001
Uric acid, umol/L	329.09 ± 98.33	341.34 ± 99.89	315.02 ± 94.66	< 0.001
Fasting blood glucose, mmol/L	8.37 ± 3.33	8.71 ± 3.24	7.98 ± 3.39	< 0.001
Alanine transaminase, U/L	26.53 ± 24.15	31.07 ± 28.27	21.45 ± 17.79	< 0.001
Aspartate aminotransferase, U/L	21.89 ± 14.05	23.89 ± 17.09	20.56 ± 19.93	< 0.001
eGFR, mL·minute^−1^·1.73m^−2^	105.29 ± 24.13	108.52 ± 23.79	101.57 ± 24.01	< 0.001
Total cholesterol, mmol/L	4.72 ± 1.21	4.80 ± 1.22	4.63 ± 1.23	0.021
Triglyceride, mmol/L	1.90 ± 1.87	2.22 ± 2.25	1.56 ± 1.47	< 0.001
High-density lipoprotein, mmol/L	1.04 ± 0.29	0.99 ± 0.25	1.08 ± 0.31	< 0.001
Low-density lipoprotein, mmol/L	2.84 ± 1.02	2.86 ± 0.99	2.81 ± 1.04	0.347
Glycosylated hemoglobin, %	10.52 ± 2.28	10.29 ± 2.12	10.79 ± 2.43	< 0.001
Fasting insulin, uU/mL	17.00 ± 27.04	15.51 ± 23.00	18.71 ± 30.98	0.051
Osteocalcin, ng/mL	11.76 ± 5.45	11.82 ± 5.45	11.69 ± 5.46	0.694
Parathyroid hormone, pg/mL	42.83 ± 22.15	45.63 ± 23.33	39.62 ± 20.25	< 0.001
Calcitonin, pg/mL	1.92 ± 2.05	1.92 ± 2.03	1.92 ± 2.07	0.976
25-hydroxyvitamin D, ng/mL	14.66 ± 5.73	14.21 ± 5.33	15.17 ± 6.13	0.005
TP1NP, ng/mL	41.90 ± 19.92	41.60 ± 19.56	42.24 ± 20.33	0.593
CTX, ng/mL	0.46 ± 0.30	0.45 ± 0.27	0.48 ± 0.32	0.069
Antihypertensive medication	458 (40.4%)	243 (40.0%)	215 (40.7%)	0.814
Antiplatelet therapy	141 (12.4%)	66 (10.9%)	75 (14.2%)	0.090
Statins	156 (13.7%)	77 (12.7%)	79 (15.0%)	0.266
Oral hypoglycemic therapy	661 (58.2%)	323 (53.2%)	338 (64.0%)	< 0.001
Insulin therapy	316 (27.8%)	150 (24.7%)	166 (31.4%)	0.012
Body composition				
Body mass index, kg/m^2^	24.17 ± 3.83	26.15 ± 3.66	21.89 ± 2.53	< 0.001
Waist circumference, cm	90.29 ± 10.12	94.42 ± 9.86	85.54 ± 8.13	< 0.001
Body fat mass, kg	18.71 ± 7.64	21.72 ± 8.18	15.26 ± 5.12	< 0.001
Soft lean mass, kg	44.20 ± 8.45	47.88 ± 8.49	39.97 ± 6.12	< 0.001
Fat free mass, kg	46.90 ± 8.91	50.77 ± 8.96	42.44 ± 6.42	< 0.001
Skeletal muscle mass, kg	25.63 ± 5.45	27.99 ± 5.49	22.91 ± 3.95	< 0.001
Percent body fat, %	27.98 ± 7.86	29.52 ± 7.97	26.22 ± 7.35	< 0.001
Muscle mass arm, kg	5.00 ± 1.31	5.57 ± 1.32	4.35 ± 0.95	< 0.001
Muscular mass trunk, kg	21.16 ± 4.11	22.89 ± 4.12	19.18 ± 3.07	< 0.001
Muscle mass leg, kg	14.08 ± 3.26	15.44 ± 3.19	12.53 ± 2.57	< 0.001
Fat mass arm, kg	2.61 ± 1.53	3.11 ± 1.78	2.03 ± 0.87	< 0.001
Fat mass trunk, kg	9.62 ± 4.14	11.29 ± 4.31	7.71 ± 2.95	< 0.001
Fat mass leg, kg	5.34 ± 1.93	6.10 ± 2.07	4.46 ± 1.28	< 0.001
Basal metabolic rate, kJ/(m^2^·h)	1382.95 ± 192.37	1466.71 ± 193.52	1286.67 ± 138.56	< 0.001
Waist-hip ratio	0.92 ± 0.06	0.93 ± 0.07	0.90 ± 0.05	< 0.001
Visceral fat area, cm^2^	91.25 ± 40.18	104.35 ± 43.33	76.20 ± 29.84	< 0.001
Body cell mass, kg	30.34 ± 5.99	32.93 ± 6.03	27.36 ± 4.34	< 0.001
BMC, kg	2.74 ± 0.52	2.96 ± 0.54	2.49 ± 0.35	< 0.001
Skeletal muscle index, kg/m^2^	6.90 ± 1.05	7.44 ± 0.96	6.27 ± 0.78	< 0.001

NAFLD, nonalcoholic fatty liver disease; Hs-CRP, highly sensitive C-reactive protein; eGFR, estimated glomerular filtration rate; TP1NP, total procollagen type I N-terminal propeptide; CTX, carboxy-terminal cross-linked telopeptide of type I collagen; BMC, bone mineral content.

**Table 2:** Characteristics of the participants according to age and sarcopenia status.

Characteristic	Elderly group (*N* = 529)			Non-elderly group (*N* = 606)		
	Non-sarcopenia *N* = 224	Sarcopenia *N* = 305	*P* value	Non-sarcopenia *N* = 383	Sarcopenia *N* = 223	*P* value
Age, yrs	7.34 ± 8.16	72.03 ± 8.21	0.019	44.05 ± 10.07	47.65 ± 9.40	< 0.001
Male	111 (49.6%)	184 (60.3%)	0.014	262 (68.4%)	180 (80.7%)	0.001
Smoking	32 (14.3%)	48 (15.7%)	0.645	92 (24.0%)	57 (25.6%)	0.671
Drinking	25 (11.2%)	29 (9.5%)	0.535	64 (16.7%)	33 (14.8%)	0.536
NAFLD	78 (34.8%)	50 (16.4%)	< 0.001	218 (56.9%)	78 (35.0%)	< 0.001
Metabolic syndrome	175 (78.1%)	207 (67.9%)	0.009	300 (78.3%)	119 (53.4%)	< 0.001
Hypertension	162 (72.3%)	196 (64.3%)	0.050	119 (31.1%)	51 (22.9%)	0.030
Osteoporosis	33 (14.7%)	45 (14.8%)	0.994	7 (1.8%)	10 (4.4%)	0.110
Fracture	15 (6.7%)	24 (7.9%)	0.610	19 (5.0%)	15 (6.7%)	0.362
CAD	22 (9.8%)	56 (18.4%)	0.006	8 (2.1%)	5 (2.2%)	0.900
Chronic kidney disease	24 (10.7%)	43 (14.4%)	0.248	11 (2.9%)	12 (5.4%)	0.119
SBP, mmHg	136.21 ± 18.67	131.54 ± 19.69	0.006	130.04 ± 16.65	127.22 ± 18.28	0.053
DBP, mmHg	80.90 ± 10.10	79.09 ± 10.61	0.048	87.82 ± 11.13	84.61 ± 11.09	0.001
White blood cell, 10^9^/L	6.47 ± 1.77	6.52 ± 2.06	0.746	6.52 ± 1.79	6.40 ± 1.79	0.418
Hemoglobin, g/L	130.38 ± 16.71	128.20 ± 15.72	0.125	144.97 ± 16.47	142.10 ± 16.03	0.037
Platelets, 10^9^/L	202.15 ± 60.47	203.45 ± 66.36	0.818	223.58 ± 60.92	225.96 ± 62.97	0.647
Hs-CRP, mg/L	4.29 ± 4.43	4.48 ± 5.21	0.652	3.41 ± 3.76	3.08 ± 4.19	0.327
Albumin, g/L	37.92 ± 3.85	38.38 ± 4.39	0.216	40.70 ± 3.58	40.08 ± 4.03	0.056
Prealbumin, mg/L	219.50 ± 55.81	206.99 ± 52.92	0.009	247.06 ± 52.73	232.37 ± 55.74	0.001
Uric acid, umol/L	324.71 ± 95.85	310.75 ± 91.49	0.090	351.06 ± 101.04	320.85 ± 98.74	< 0.001
FBG, mmol/L	7.87 ± 2.92	7.66 ± 3.46	0.455	9.20 ± 3.31	8.42 ± 3.24	0.005
ALT, U/L	24.37 ± 18.95	20.01 ± 17.09	0.007	34.95 ± 31.85	23.43 ± 18.57	< 0.001
AST, U/L	22.74 ± 14.24	20.22 ± 12.01	0.028	24.57 ± 18.54	19.37 ± 12.46	< 0.001
eGFR, mL·minute^−1^·1.73m^−2^	88.54 ± 20.04	90.51 ± 19.21	0.251	120.21 ± 17.09	116.69 ± 21.58	0.027
TC, mmol/L	4.47 ± 1.17	4.44 ± 1.20	0.829	4.99 ± 1.20	4.88 ± 1.22	0.290
Triglyceride, mmol/L	1.63 ± 1.05	1.35 ± 0.71	0.001	2.56 ± 2.65	1.85 ± 2.06	< 0.001
HDL, mmol/L	1.06 ± 0.29	1.09 ± 0.28	0.225	0.95 ± 0.22	1.07 ± 0.35	< 0.001
LDL, mmol/L	2.66 ± 1.00	2.68 ± 1.06	0.838	2.98 ± 0.97	2.98 ± 0.99	0.967
HbA1c, %	9.68 ± 2.21	10.47 ± 2.49	< 0.001	10.64 ± 1.98	11.23 ± 2.26	0.001
Fasting insulin, uU/mL	14.81 ± 20.59	20.10 ± 31.97	0.021	15.92 ± 24.32	16.82 ± 29.53	0.686
Osteocalcin, ng/mL	12.08 ± 6.35	11.02 ± 5.60	0.043	11.67 ± 4.84	12.61 ± 5.14	0.024
PTH, pg/mL	47.44 ± 25.60	39.21 ± 19.54	< 0.001	44.58 ± 21.86	40.17 ± 21.22	0.016
Calcitonin, pg/mL	1.92 ± 2.13	1.68 ± 1.84	0.165	1.91 ± 1.98	2.25 ± 2.31	0.056
25(OH)D, ng/mL	14.96 ± 6.16	15.56 ± 5.99	0.261	13.76 ± 4.73	14.64 ± 6.28	0.071
TP1NP, ng/mL	41.79 ± 19.73	39.30 ± 18.03	0.132	41.49 ± 19.48	46.25 ± 22.55	0.006
CTX, ng/mL	0.40 ± 0.22	0.40 ± 0.22	0.957	0.47 ± 0.29	0.58 ± 0.40	< 0.001
Antihypertensive medication	153 (68.3%)	174 (57.0%)	0.008	90 (23.5%)	41 (18.4%)	0.140
Antiplatelet therapy	48 (21.4%)	68 (22.3%)	0.812	18 (4.7%)	7 (3.1%)	0.352
Statins	53 (23.7%)	66 (21.6%)	0.582	24 (6.3%)	13 (5.8%)	0.829
Oral hypoglycemic therapy	156 (69.6%)	231 (75.7%)	0.118	167 (43.6%)	107 (48.0%)	0.296
Insulin therapy	81 (36.2%)	117 (38.4%)	0.605	69 (18.0%)	49 (22.0%)	0.235
Body composition						
BMI, kg/m^2^	25.16 ± 3.23	21.88 ± 2.60	< 0.001	26.74 ± 3.78	21.90 ± 2.45	< 0.001
WC, cm	93.17 ± 9.37	86.13 ± 8.32	< 0.001	95.15 ± 10.08	84.74 ± 7.80	< 0.001
Body fat mass, kg	20.15 ± 7.26	15.47 ± 5.11	< 0.001	22.64 ± 8.55	14.97 ± 5.14	< 0.001
Soft lean mass, kg	43.66 ± 6.87	38.14 ± 5.68	< 0.001	50.35 ± 8.38	42.83 ± 5.82	< 0.001
Fat free mass, kg	46.32 ± 7.22	40.52 ± 5.95	< 0.001	53.38 ± 8.86	45.06 ± 6.11	< 0.001
SMM, kg	25.09 ± 4.31	21.64 ± 3.59	< 0.001	29.68 ± 5.39	24.65 ± 3.76	< 0.001
Percent body fat, %	29.86 ± 8.83	27.40 ± 7.62	0.001	29.31 ± 7.43	24.62 ± 6.65	< 0.001
Muscle mass arm, kg	4.88 ± 1.12	4.10 ± 0.90	< 0.001	5.97 ± 1.27	4.70 ± 0.92	< 0.001
MMT, kg	20.66 ± 3.46	18.28 ± 2.86	< 0.001	24.20 ± 3.92	20.41 ± 2.92	< 0.001
Muscle mass leg, kg	14.16 ± 3.05	11.73 ± 2.42	< 0.001	16.18 ± 3.04	13.62 ± 2.36	< 0.001
Fat mass arm, kg	2.89 ± 1.39	2.12 ± 0.89	< 0.001	3.24 ± 1.96	1.92 ± 0.84	< 0.001
Fat mass trunk, kg	10.32 ± 4.10	7.82 ± 2.93	< 0.001	11.85 ± 4.34	7.57 ± 2.98	< 0.001
Fat mass leg, kg	5.81 ± 1.84	4.49 ± 1.28	< 0.001	6.28 ± 2.17	4.41 ± 1.28	< 0.001
BMR, kJ/(m^2^·h)	1370.44 ± 155.92	1245.34 ± 128.48	< 0.001	1523.01 ± 191.33	1343.19 ± 131.96	< 0.001
Waist-hip ratio	0.92 ± 0.07	0.90 ± 0.05	< 0.001	0.94 ± 0.06	0.90 ± 0.05	< 0.001
Visceral fat area, cm^2^	101.42 ± 42.89	80.53 ± 31.36	< 0.001	106.07 ± 43.54	70.27 ± 26.55	< 0.001
Body cell mass, kg	29.75 ± 4.74	25.96 ± 3.94	< 0.001	34.80 ± 5.92	29.27 ± 4.13	< 0.001
BMC, kg	2.72 ± 0.42	2.41 ± 0.33	< 0.001	3.10 ± 0.56	2.60 ± 0.35	< 0.001
SMI, kg/m^2^	7.06 ± 0.90	6.05 ± 0.73	< 0.001	7.66 ± 0.92	6.58 ± 0.74	< 0.001

NAFLD, nonalcoholic fatty liver disease; CAD, coronary artery disease; SBP, systolic blood pressure; DBP, diastolic blood pressure; Hs-CRP, highly sensitive C-reactive protein; FBG, fasting blood glucose; ALT, alanine transaminase; AST, aspartate aminotransferase; eGFR, estimated glomerular filtration rate; TC, total cholesterol; HDL, high-density lipoprotein; LDL, low-density lipoprotein; HbA1c, glycosylated hemoglobin; PTH, parathyroid hormone; 25(OH)D, 25-hydroxyvitamin D; TP1NP, total procollagen type I N-terminal propeptide; CTX, carboxy-terminal cross-linked telopeptide of type I collagen; BMI, body mass index; WC, waist circumference; SMM, skeletal muscle mass; MMT, muscular mass trunk; BMR, basal metabolic rate; BMC, bone mineral content; SMI, skeletal muscle index.

### Correlation coefficients for BMC

The correlations between BMC and all the variables and characteristics are presented in Tables 3. The BMC level was positively correlated with diastolic blood pressure, hemoglobin, albumin, prealbumin, uric acid, fasting blood glucose, alanine transaminase, aspartate aminotransferase, eGFR, triglyceride, calcitonin and all body composition indicators except percent body fat. This measurement was also positively correlated with a history of smoking, drinking, and nonalcoholic fatty liver disease. The BMC level was negatively correlated with age, gender, HDL, and percent body fat. This measurement was also negatively correlated with a history of sarcopenia, hypertension, osteoporosis and CAD. In addition, the BMC level was negatively correlated with the use of antihypertensive medication, antiplatelet therapy, statins, oral hypoglycemic therapy, and insulin therapy.

**Table 3:** Spearman correlation coefficients for BMC.

Variable	BMC	*P* value
Age	−0.431	< 0.001
Gender	−0.602	< 0.001
Smoking	0.240	< 0.001
Drinking	0.165	< 0.001
Sarcopenia	−0.448	< 0.001
Nonalcoholic fatty liver disease	0.180	< 0.001
Hypertension	−0.088	0.003
Osteoporosis	−0.234	< 0.001
Coronary artery disease	−0.135	< 0.001
Diastolic blood pressure	0.192	< 0.001
Hemoglobin	0.414	< 0.001
Albumin	0.165	< 0.001
Prealbumin	0.253	< 0.001
Uric acid	0.268	< 0.001
Fasting blood glucose	0.154	< 0.001
Alanine transaminase	0.203	< 0.001
Aspartate aminotransferase	0.063	0.035
Estimated glomerular filtration rate	0.298	< 0.001
Triglyceride	0.172	< 0.001
High-density lipoprotein	−0.315	< 0.001
Calcitonin	0.282	< 0.001
Antihypertensive medication	−0.119	< 0.001
Antiplatelet therapy	−0.066	0.027
Statins	−0.079	0.008
Oral hypoglycemic therapy	−0.131	< 0.001
Insulin therapy	−0.106	< 0.001
Body mass index	0.440	< 0.001
Waist circumference	0.438	< 0.001
Body fat mass	0.198	< 0.001
Soft lean mass	0.914	< 0.001
Fat free mass	0.919	< 0.001
Skeletal muscle mass	0.907	< 0.001
Percent body fat	−0.238	< 0.001
Muscle mass arm	0.854	< 0.001
Muscular mass trunk	0.857	< 0.001
Muscle mass leg	0.880	< 0.001
Fat mass arm	0.082	0.006
Fat mass trunk	0.230	< 0.001
Fat mass leg	0.179	< 0.001
Basal metabolic rate	0.919	< 0.001
Waist-hip ratio	0.194	< 0.001
Visceral fat area	0.085	0.004
Body cell mass	0.907	< 0.001
Skeletal muscle index	0.845	< 0.001

### Discriminative ability of BMC and bone metabolic markers for sarcopenia

The ROC curve of BMC level as a predictive factor for sarcopenia highlighted the medium ability of BMC to predict sarcopenia. The area under the curve (AUC) was 0.760 (95%CI 0.732–0.787, *P* < 0.001). For BMC, the optimal cutoff was 2.86 kg (sensitivity 55.8%, specificity 86.0%). The optimal PTH cutoff for predicting sarcopenia was 38.45pg/mL, yielding a specificity of 56.8% and sensitivity of 58.2%. The AUC was 0.591 (95%CI 0.557–0.624, *P* < 0.001). The ROC curve of BMC and bone metabolic markers as predictive factors for sarcopenia in the total, elderly, and non-elderly subjects were showed in Figures 1–3.

**Figure 1:**
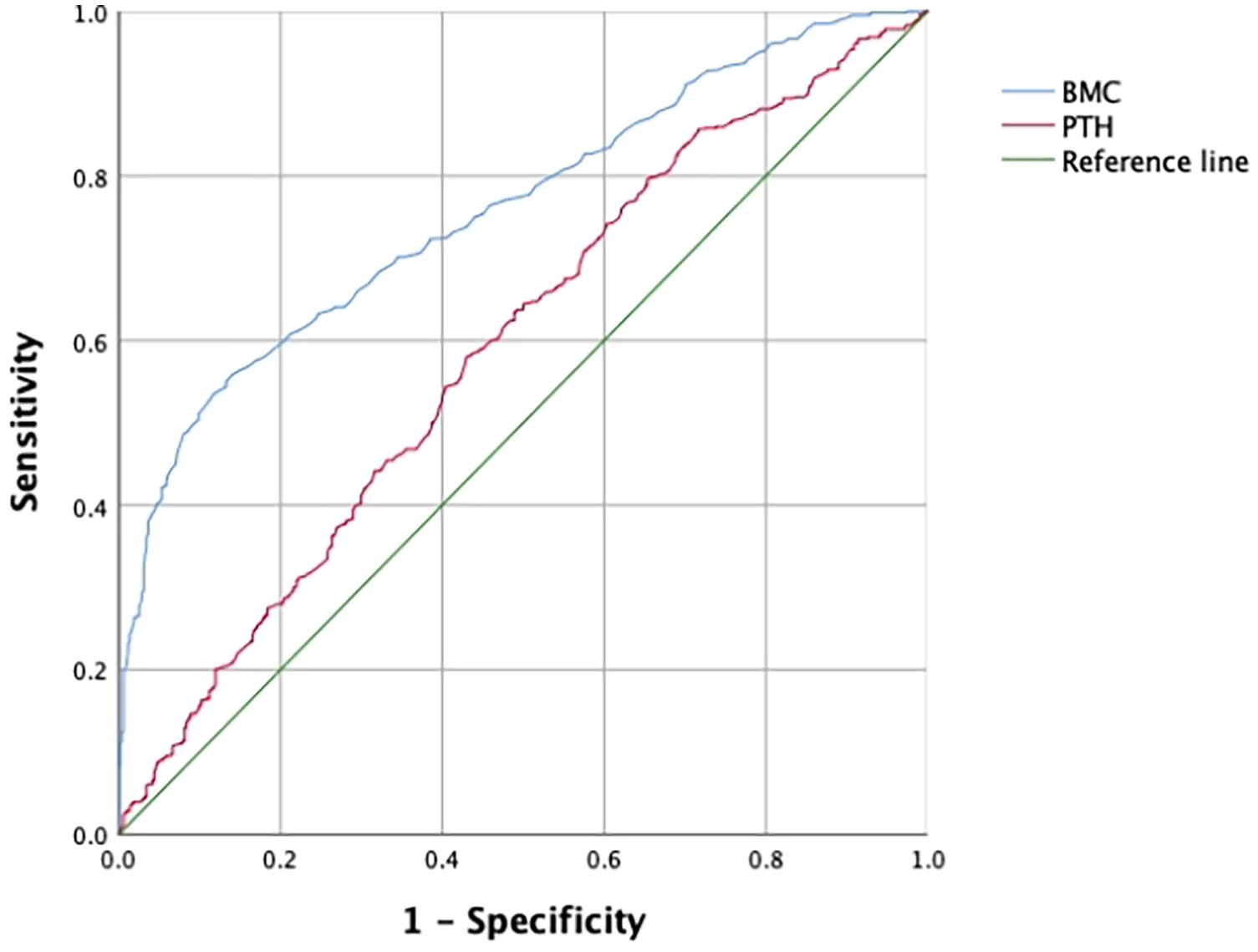
ROC curve based on a univariate model examining the power of BMC and PTH to predict sarcopenia. The optimal cut-off level for BMC to predict sarcopenia was found to be 2.86 kg, with a specificity of 86.0% and a sensitivity of 55.8%. The area under the curve was 0.760 (95%CI 0.732-0.787, *P* < 0.001). The optimal cut-off level for PTH to predict sarcopenia was found to be 38.45pg/mL, with a specificity of 56.8% and a sensitivity of 58.2%. The area under the curve was 0.591 (95%CI 0.557-0.624, *P* < 0.001).

**Figure 2:**
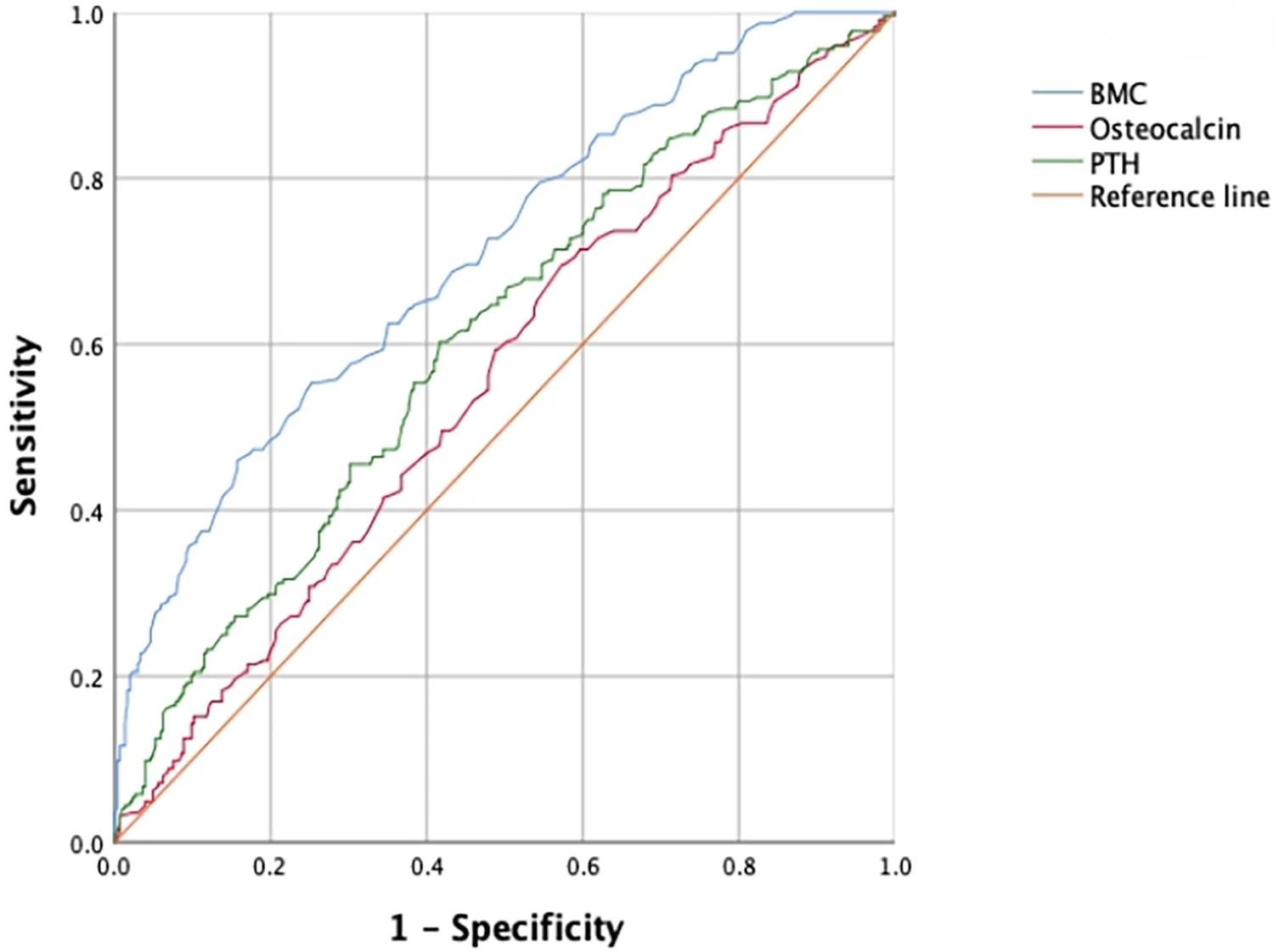
ROC curve based on a univariate model examining the power of BMC, osteocalcin and PTH to predict sarcopenia in elderly subjects. The optimal cut-off level for BMC to predict sarcopenia in elderly subjects was found to be 2.74 kg, with a specificity of 84.3% and a sensitivity of 46.0%. The area under the curve was 0.709 (95%CI 0.665-0.753, *P* < 0.001). The optimal cut-off level for osteocalcin to predict sarcopenia in elderly subjects was found to be 9.05 ng/mL, with a specificity of 42.6% and a sensitivity of 69.6%. The area under the curve was 0.557 (95%CI 0.508-0.607, *P* = 0.024). The optimal cut-off level for PTH to predict sarcopenia in elderly subjects was found to be 38.45pg/mL, with a specificity of 58.4% and a sensitivity of 60.3%. The area under the curve was 0.606 (95%CI 0.558-0.655, *P* < 0.001).

**Figure 3:**
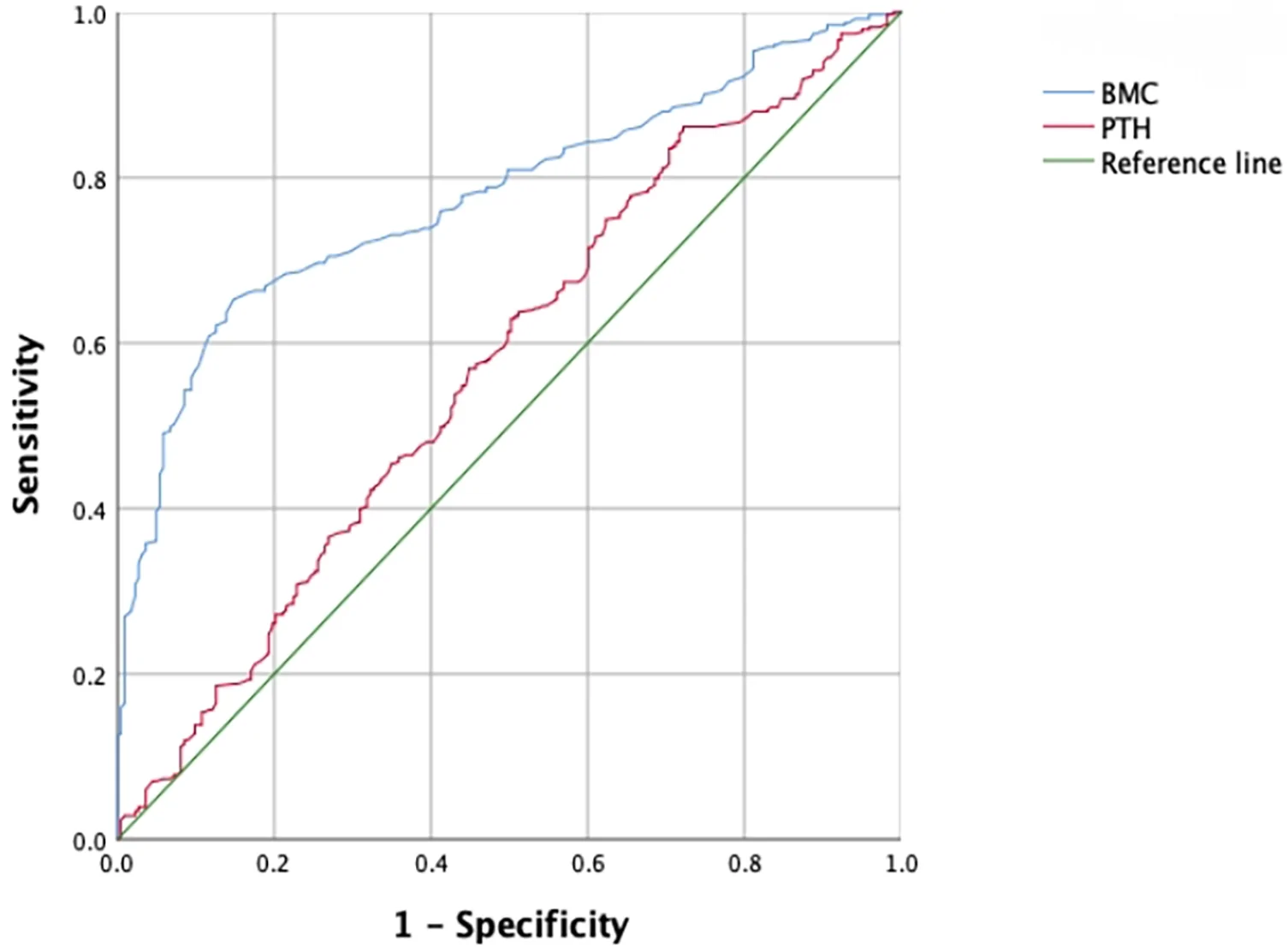
ROC curve based on a univariate model examining the power of BMC and PTH to predict sarcopenia in non-elderly subjects. The optimal cut-off level for BMC to predict sarcopenia in non-elderly subjects was found to be 2.91 kg, with a specificity of 85.2% and a sensitivity of 65.3%. The area under the curve was 0.777 (95%CI 0.740-0.813, *P* < 0.001). The optimal cut-off level for PTH to predict sarcopenia in non-elderly subjects was found to be 26.60pg/mL, with a specificity of 22.8% and a sensitivity of 86.2%. The area under the curve was 0.572 (95%CI 0.525-0.620, *P* = 0.003).

### Effects of BMC < optimal cut-off level on sarcopenia

The effects of BMC < optimal cut-off level on sarcopenia in the total, elderly, and non-elderly groups used the Multivariable Logistic Regression model with the forward stepwise method were showed in Tables 4. With the higher BMC level as the reference, a BMC level < 2.86 kg (OR=4.994, 95%CI 3.130–7.967, *P* < 0.001) remained associated with an increased risk of sarcopenia after adjustment for variables that were found to be statistically significant in the univariate analysis. When patients were divided into elderly and non-elderly groups, BMC < optimal cut-off level was also associated with an increased risk of sarcopenia after adjustment for variables that were found to be statistically significant in the univariate analysis (OR=7.025, 95% CI 3.500–14.098, *P* < 0.001 in the elderly group and OR=6.809, 95% CI 3.294–14.075, *P* < 0.001 in the non-elderly group).

**Table 4:** Effects of BMC < optimal cut-off level on sarcopenia in multivariable logistic regression analysis.

BMC < optimal cut-off level	Model 1	P	Model 2	P	Model 3	P	Model 4	P	Model 5	P
	(OR, 95%CI)		(OR, 95%CI)		(OR, 95%CI)		(OR, 95%CI)		(OR, 95%CI)	
Whole	7.760 (5.788, 10.406)	<0.001	16.377 (11.265, 23.810)	<0.001	13.866 (9.361, 20.539)	<0.001	14.060 (9.465, 20.888)	<0.001	4.994 (3.130, 7.967)	<0.001
Elderly group	4.558 (3.040, 6.833)	<0.001	13.337 (7.592, 23.428)	<0.001	14.499 (7.957, 26.422)	<0.001	14.996 (8.172, 27.519)	<0.001	7.025 (3.500, 14.098)	<0.001
Non-elderly group	10.823 (7.073, 16.559)	<0.001	33.769 (19.892, 57.326)	<0.001	29.129 (16.721, 50.746)	<0.001	29.129 (16.721, 50.746)	<0.001	6.809 (3.294, 14.075)	<0.001

Model 1: crude model.

Model 2: adjusted for general situation including age and gender.

Model 3: adjusted for Model 2 and laboratory examination indicators including white blood cell, hemoglobin, platelets, highly sensitive C-reactive protein, albumin, prealbumin, uric acid, fasting blood glucose, alanine transaminase, aspartate aminotransferase, estimated glomerular filtration rate, total cholesterol, triglyceride, high-density lipoprotein, low-density lipoprotein, glycosylated hemoglobin, fasting insulin, osteocalcin, parathyroid hormone, calcitonin, 25-hydroxyvitamin D, total procollagen type I N-terminal propeptide, carboxy-terminal cross-linked telopeptide of type I collagen, systolic blood pressure and diastolic blood pressure.

Model 4: adjusted for Model 3 and underlying diseases and medication history including nonalcoholic fatty liver disease, metabolic syndrome, hypertension, osteoporosis, fracture, coronary artery disease, chronic kidney disease, antihypertensive medication, antiplatelet therapy, statins, oral hypoglycemic therapy and insulin therapy.

Model 5: adjusted for Model 4 and body composition indicators including body mass index, waist circumference, and percent body fat, etc., while excluding those body composition indicators that are related to the diagnosis of sarcopenia.

Variables with *P* < 0.1 in the univariate analyses within each of the three groups were respectively entered into the corresponding multivariable models.

## Discussion

Our findings were obtained using the recently published AWGS 2025 diagnostic criteria, which represent the current consensus for sarcopenia diagnosis in Asian populations (18). In our study, the prevalence of sarcopenia in T2DM patients was found to be 46.5%, which is higher than previously reported figures, possibly because all the patients included were inpatients, and the proportion of the elderly was even higher. Our study showed that among patients with T2DM, the sarcopenia group exhibited a lower BMC level than the non-sarcopenia group. This finding raises the hypothesis that BMC may exert a protective effect on musculoskeletal health, warranting further investigation. The BMC level was positively correlated with diastolic blood pressure, hemoglobin, albumin, prealbumin, uric acid, fasting blood glucose, alanine transaminase, aspartate aminotransferase, eGFR, triglyceride, calcitonin and all body composition indicators except percent body fat. In addition, BMC levels were also negatively correlated with a history of sarcopenia, hypertension, osteoporosis and CAD. In this study, BMC exhibited good discriminative performance (high specificity but low sensitivity) in our population (area under the ROC curve=0.760); however, independent validation in external cohorts is needed before they can be adopted as clinically definitive thresholds. Lastly, in the Multivariable Logistic Regression analysis with the higher BMC level as the reference, a BMC level < 2.86 kg remained significantly associated with sarcopenia irrespective of the subgroup of age (including the elderly group and non-elderly group), highlighting the potential unique association between BMC and sarcopenia.

T2DM subjects face a higher sarcopenia risk (19–21), and sarcopenia itself, along with other factors, contributes to bone fragility in this population (22). BMC was analyzed instead of the more commonly used BMD as it is a better surrogate measure of bone strength and allows for comparison among and within participants—due to the utilization of an areal form of density, BMD is subject to bias when comparing bones of different sizes (23–25). Similar to our study on T2DM, He Q, et al. found that compared to nonsarcopenic diabetic controls, sarcopenic diabetic patients were older, had lower BMI, and worse nutritional status with an unbalanced nutrition intake and demonstrated deteriorated glucose metabolism, decreased renal function, and a reduction in muscle quality and skeletal muscle mass with a greater likelihood of osteoporosis (26). Moreover, Xu K, et al. found that the BMD of the hip and femoral neck in T2DM patients is related to sarcopenia-related indicators and represents an independent protective factor for sarcopenia, demonstrating that T2DM patients in China complicated with sarcopenia had a higher tendency to suffer from osteoporosis compared with those without sarcopenia (27). And Xiao T, et al. revealed a relatively high prevalence of osteosarcopenia in elderly T2DM patients, with significant associations identified between low fasting plasma glucose, male gender, and low Appendicular Skeletal Muscle Mass Index as major risk factors (28). Different from these, a previous study in 2025 revealed that T2DM had different paradoxical relationships on bone mass, and skeletal muscle mass and function. Although hyperinsulinemia in T2DM might have anabolic effects on bone, it has negative associations with the neuromuscular system (sarcopenia), possibly explaining the increased fall and fracture risks (29). Over the last decade, despite several studies demonstrating an association between bone fragility and sarcopenia in T2DM patients, few have focused on the relationship between BMC and sarcopenia among these patients as our study.

Sarcopenia has been described as another notable complication of diabetes in addition to microvascular and macrovascular complications. Meanwhile, diabetes is also considered an independent risk factor for fragility fractures (30). Both muscle and bone originate from mesenchymal stem cells, and the developmental homology makes them closely related. Muscle and bone are not only adjacent to each other in anatomical position but also regulated by common genes, with common paracrine and endocrine mechanisms and similar molecular signaling pathways (31). Bone and muscle can secrete a variety of biochemical factors, named myokines and osteokines respectively, which perform an important role in the interaction between muscle and bone. The cells in bone tissues can secrete multiple osteokines including osteocalcin (OCN), prostaglandin E2 (PGE2), fibroblast growth factor-23 (FGF23), receptor activator of nuclear factor-*κ*B (NF-*κ*B) ligand (RANKL) and so on. OCN is synthesized and secreted by osteoblasts and binds to G protein coupled receptor C family 6 group A (GPRC6A). OCN increases muscle mass and regulates muscle function via the GPRC6A/AMPK/mTOR/S6 kinase pathway. GPRC6A gene knock out mice have decreased muscle mass, while the muscle mass is increased in embryonic stem phosphatase gene knockout mice, indicating the promoting effect of OCN on muscle mass and muscle function (32). PGE2 secreted by osteocytes is 1,000 times higher than that secreted by myocytes. The excessive PGE2 in bone cells interacts with damaged muscles, which facilitates muscle regeneration and repair (33). The high level of FGF23 in serum negatively regulates the synthesis of 1,25-dihydroxyvitamin D [1,25 (OH) D] (34), triggering cardiovascular diseases, weakness, disability, and other adverse consequences. In addition to the paracrine interaction between muscle and bone, they also have common signaling pathway regulation. The NF-*κ*B pathway is one of the most important signaling pathways in muscle, and its activation leads to skeletal muscle atrophy. Some scholars believe that NF-*κ*B activation-induced muscle atrophy may be related to the increase of inflammatory factors such as TNF-α and IL-6, and blocking this pathway may represent a promising target to treat muscle atrophy. In bone tissue, the NF-*κ*B signaling pathway participates in corticosteroid-induced osteocyte apoptosis and mediates RANKL-induced osteoclast formation, differentiation, and maturation. Moreover, the Wnt/*β*-catenin signaling pathway is deemed as one of the signaling pathways that simultaneously regulates bone and muscle metabolism. Wnt signal can regulate osteoblast activity, muscleformation, and regeneration in the embryonic stage (35, 36).

To the best of our knowledge, this study is the first to investigate the relationship between BMC and sarcopenia in patients referred for T2DM. Remarkably, lower BMC level was associated with an increased risk of sarcopenia irrespective of the subgroup of age (including elderly group and non-elderly group) in our study. These findings suggest that BMC may discriminate sarcopenia severity and raise the possibility that its upregulation could have therapeutic potential for diabetic patients with sarcopenia, though this remains to be explored.

### Study limitations

This study has several limitations. Firstly, as a single-center, cross sectional study, this study failed to determine the causal relationship between BMC and sarcopenia. Secondly, although we adjusted demographic variables, clinical data, laboratory values, and information on patients’ past medical history and other factors in the statistical analysis, some important questionnaire contents were still not considered, such as family economic level, dietary habits, exercise frequency, sunshine time, etc. Thirdly, our study subjects were limited to the Chinese population, and the conclusions may have certain disparities among people of different races and latitudes. Fourthly, only baseline measurements of BMC were available. Therefore, it was not possible to assess changes in the BMC levels over time or the implications of these changes.

## Conclusion

The BMC levels exhibit an inverse relationship with sarcopenia. These findings suggest that BMC may help differentiate sarcopenia severity in patients with T2DM, and raise the hypothesis that its upregulation could confer musculoskeletal benefits and serve as a candidate therapeutic target—although this requires further investigation.

## Data Availability

The raw data supporting the conclusions of this article will be made available by the authors, without undue reservation.
